# Overview of Cellular Immunotherapy for Patients with Glioblastoma

**DOI:** 10.1155/2010/689171

**Published:** 2010-10-04

**Authors:** Elodie Vauleon, Tony Avril, Brigitte Collet, Jean Mosser, Véronique Quillien

**Affiliations:** ^1^Département de Biologie, Centre Eugène Marquis, Rue de la bataille Flandres Dunkerque, CS44229, 35042 Rennes cedex, France; ^2^UMR6061 CNRS, Université de Rennes 1, IFR 140, CS34317, 35043 Rennes cedex, France

## Abstract

High grade gliomas (HGG) including glioblastomas (GBM) are the most common and devastating primary brain tumours. Despite important progresses in GBM treatment that currently includes surgery combined to radio- and chemotherapy, GBM patients' prognosis remains very poor. Immunotherapy is one of the new promising therapeutic approaches that can specifically target tumour cells. Such an approach could also maintain long term antitumour responses without inducing neurologic defects. Since the past 25 years, adoptive and active immunotherapies using lymphokine-activated killer cells, cytotoxic T cells, tumour-infiltrating lymphocytes, autologous tumour cells, and dendritic cells have been tested in phase I/II clinical trials with HGG patients. This paper inventories these cellular immunotherapeutic strategies and discusses their efficacy, limits, and future perspectives for optimizing the treatment to achieve clinical benefits for GBM patients.

## 1. Introduction

 Glioblastoma is the most common primary brain tumour in humans [1] and has the most severe prognosis [2, 3]. Despite improved surgical management and a multimodal treatment of concomitant radiotherapy and chemotherapy, followed by adjuvant chemotherapy with temozolomide (TMZ) [4], the prognosis remains poor with median survival of less than 15 months. Research has thus actively focused on testing new therapeutic approaches, including immunotherapy.

 Until recently, it was generally assumed that immune reactions do not occur in the brain because of the blood-brain barrier (BBB) and the specific features of the brain such as the absence of conventional lymphatic vessels or the low level of circulating T cells. It is now known however that the central nervous system maintains a two-way communication network with the immune system. Infectious or experimental autoimmune encephalomyelitis animal models allow better understanding of how the immune system operates in the brain.

 Under physiological conditions, the brain contains several different immune cell populations. Microglial cells, which most likely arise from hematopoietic cells, colonize the central nervous system during embryonic development and come to account for 5 to 20% of cells in the central nervous system. Macrophages and dendritic cells (DC) which arise from monocytes circulating in the blood stream are found in perivascular zones, the choroid plexuses, and the meninges. Microglial cells constitute the first line of defense for the brain. They migrate toward inflammatory zones and, after activation, possess phagocytic properties and synthesize several types of cytokines and chemokines enabling the recruitment of other immune cells [5]. Adaptive immune reactions are initiated in cervical nodes. The T cells activated in these nodes present a particular phenotype with an overexpression of *α*4/*β*7 integrins and therefore exhibit tropism for the brain [6]. At the present time, the way in which antigens are transported from the brain to the cervical nodes remains unclear. It has been shown in animal models of brain tumours that antigen presenting cells (APC) exit in the brain parenchyma by migrating along the external capsule to reach the cervical nodes [7]; the drainage system is nevertheless different in rodents and humans. In humans, it is known that brain antigens reach the cervical nodes [8], although whether they arrive associated with APC or not remains to be elucidated.

 Due to the complex cellular organisation of brain capillary vessels composed of endothelial cells with tight junctions and associated pericytic and astrocytic cells [9, 10], the BBB contributes to the selective entry of immune cells from the periphery into the brain parenchyma. In particular, the multistep model of "rolling/activation/adhesion/transmigration" through the endothelial wall has been proposed to control the arrival of activated T cells into the brain [9, 10]. This mechanism is finely regulated by multiple molecular interactions involving adhesion molecules (i.e *α*4/*β*7 integrins) and chemokines such as CXCR3 [9, 10]. However, in GBM patients, the BBB appears to be disorganised with a asymmetric structure of brain capillaries into the tumour, a dysfunction of tight junctions between endothelial cells, and a decrease in BBB-associated pericytes [9, 11, 12]. In this context, rules that control the trafficking of effector T cells into the tumour site might be completely different. 

 Different models have been proposed to better understand the effector phase of the immune response in the brain. For example, following a nasal infection with a herpes simplex virus, the first observation is the arrival of macrophages and a few neutrophils, then a few NK and T cells, which will become predominant [13]. Lymphocyte migration was followed in a recent study with injections of CD4+ lymphocytes directed against myelin proteins in a model of autoimmune encephalitis: after arriving in the subarachnoid spaces (in leptomeningeal vessels), the lymphocytes migrated along the internal wall of the vessels. After diapedesis, they migrated along the external walls and if they encountered APC (macrophages or DC) presenting myelin antigens, they were then reactivated, synthesized numerous cytokines, and penetrated the brain parenchyma [14]. The brain environment plays a key role for the local expansion of CD8+ lymphocytes. In a glioma mouse model, after they arrived to the brain, CD8+ T cells can proliferate and further differentiate with enhanced IFN*γ* and granzyme B expression. These conditioned CD8+ T cells express *α*E*β*7 integrins which enhance their retention into the brain [15]. This induced expression of *α*E*β*7 integrins could be due to the presence of TGF*β*. In an infection model, a secondary expansion of CD8+ T cells into the brain was observed and was due to the presence of DC *in situ* [16]. 

 An adaptive immune response implies antigen recognition. Our team has demonstrated, in a series of 47 GBM, the frequent overexpression of four tumour antigens: IL13R*α*2, EGFRvIII, gp100, and TRP2 [17], which are known to induce immune reactions. Other antigens associated with GBM have been described including EphA2, survivin, WT1, SOX2, SOX11, MAGE1, AIM2, and SART1 [18]. T cells directed against IL13R*α*2 and EphA2 have been demonstrated in the peripheral blood mononuclear cells (PBMCs) of a long surviving patient with anaplastic astrocytoma, showing that a spontaneous immune reaction can occur in HGG [19]. While T-cell infiltration is not observed in the normal brain, it can be observed in glioma. Mittelbronn et al. used an immunohistochemistry technique to demonstrate CD8 expression in a few cells (less than 0.1% of the total cell mass) in a series of GBM [20]. In four GBM, Barcia et al. found that CD8+ T cells are preferentially located in the brain parenchyma and in proximity to vessels while CD4+ T cells (less numerous than CD8+ cells in 3 of the 4 cases) are not found in the parenchyma. In addition, these lymphocytes can express granzyme and form synapses with tumour cells. A few granzyme-expressing NK cells were also found [21]. In the latest contribution, another team used flow cytometry to demonstrate that 0.1 to 3.2% of cells found in tumour tissues express CD3. Half of these cells also express CD56 and are for the majority CD4+. However, these unusual CD4+ CD56+ cells, preferentially found in GBM, do not express CD1d or V*α*24 TCR chains that are typical markers for NKT cells [22]. Overall, these results suggest that immune reactions are occurring in primary brain tumours, but are largely ineffective as shown by the inevitable recurrence of the disease.

 There could be two ways of using immunotherapy for brain tumours. The first is the “active immunotherapy” designed to boost the patient's native immune response. The second is the “passive immunotherapy” where *in vitro* activated immune cells or specific molecules (e.g. antibodies) directly targeting tumour cells are injected. This paper is focused on immunotherapy trials based on cell therapy, exclusively published until may 2010 and inventoried in MEDLINE accessed by the NCBI Pubmed database. All trials with GBM patients (included trials with GBM and other HGG) were selected.

## 2. Adoptive Immunotherapy

 In adoptive immunotherapy, immune cells activated *ex vivo* are administrated to the tumour-bearing patient. The activated cells are either injected directly into the tumour cavity or intravenously. The first types of cells used for gliomas were lymphocyte-activated killer (LAK) cells [23–34]. LAK cells are generally obtained by cultivating peripheral lymphocytes in the presence of IL2 (±lectins) yielding populations with different sets of T cells and NK cells with cytolytic properties not specifically directed against tumour cells. Cytotoxic T lymphocytes (CTL) can also be used. CTL can be generated by *ex vivo* antigenic stimulation of PBMC. For gliomas, autologous tumour cells (ATC) are generally used as antigen source [35–37]. Allogeneic CTL stimulated by the patient's own lymphocytes have also been tested [38]. After amplification in the presence of IL2, CTL can be obtained from tumour infiltrating lymphocytes (TIL), as was done in one study [39]. Another approach was to collect lymphocytes from lymph nodes or PBMC after peripheral injection of irradiated ATC and GM-CSF, stimulating them *in vitro* then reinjecting them [40–44]. 

### 2.1. Adoptive Immunotherapy Using LAK

Twelve trials treating HGG with LAK have been reported in the literature (Table 1). One pilot study [28], six phase I trials [23–26, 29, 31], and five phase I/II trials [27, 30, 32–34], including 211 patients. In all of these studies except one, the patients were included when their disease relapsed. LAK were administered intracerebrally and delivered into the operative cavity or directly into the lesion. Intrathecal administration was used in one patient. Number of injected cells ranged from 1 × 10^6^ to 1 × 10^10^ LAK, generally associated with IL2 doses to the order of 10^9^ units per injection. One to fifteen injections were given per patient.

 Neurological toxicity was observed in six of the nine trials reporting this factor. The patients presented brain edema or symptoms of aseptic meningitis. Hypereosinophilia was detected in the cerebrospinal fluid or in the peripheral blood in two patients [37] and infiltrations of mononuclears and eosinophils were found in nine of the eleven reoperations and in all three autopsies a regional eosinophilia being associated with increased survival [30, 32]. 

 To assess the efficacy of the treatment, most of the published trials reported in this paper used radiological response criteria: partial response (PR) defined as more than 50% radiographic reduction in tumour volume; minor response (MR) defined as 50 to 25% reduction; and stable disease (SD) defined as less than 25% reduction or less than 20% progression of tumour volume; finally, progressive disease (PD) defined as more than 20% progression in tumour volume or a new lesion. Radiological response reached MR at least once in nine of the twelve trials reporting the radiological response. Combining all reports in the literature, there were 5 CR (complete response) (3 GBM), 13 PR (8 GBM), and 6 SD (6 GBM) in a total of 118 patients. Correlation between the clinical results and radiological response has not been significant [27]. 

 In some trials, median survival for vaccinated GBM patients was higher than the one observed for control groups [30, 32, 33]. For the only trial on newly diagnosed GBM, median overall survival (OS) was 20.5 months, with 75% of patients alive at one year. In this study, a positive correlation was demonstrated between the number of LAK injected and survival as well as a negative correlation between corticosteroid therapy and survival [34].

### 2.2. Adoptive Immunotherapy Using Other Cell Types

Five trials testing adoptive immunotherapy for HGG have used CTL obtained from PBMC or TIL in 30 patients (Table 2). These were four phase I trials using CTL [35–38] and one pilot study using TIL [39]. In all five trials, the immune cells were injected intracerebrally. Three other phase I trials [40–42] and two pilot studies [43, 44] used CTLs obtained from lymph nodes or PBMCs after intradermal vaccination in a combination strategy using both active and adoptive immunotherapy in 65 patients. The immune cells were injected intravenously, or associated with intracarotid infusion.

 In the ten published studies, the number of injections ranged from 1 to 13. The number of cells injected varied from 3 × 10^7^ to 10 × 10^10^. IL-2 injections were associated in four trials. Delayed type hypersensitivity (DTH) was observed in reaction to the tumour cells at the point of injection in three of the five trials associating vaccination with adoptive therapy.

 Tolerance was satisfactory in all the published trials with no reported grade III/IV events, even with intravenous injections.

 Clinically, the ten published trials have reported at least a few cases of SD (*n* = 18) as well as 28 PR and 3 CR among 95 patients treated. Four studies have even been able to demonstrate a benefit in survival. One study failed to find any PR but reported disease-free survival ≥ 8 months in 7 of 15 patients treated, including one patient with > 40 months survival [40]. In one trial, there was a correlation between clinical response and the predominant CD8+ phenotype of T cells present into the vaccines [43]. A correlation between clinical response and immune response revealed by DTH response to autologous tumours was also observed [43, 44]. 

 In the pilot study using autologous TIL, the cytotoxic activity of the vaccines tested *in vitro* against autologous tumours varies depending to the patients and was not correlated to the clinical outcome [39]. However, it is interesting to note that the only patient with CR contained a distinct population of CD8+CD56+ cells (around 20%). As TIL obtained from other patients with PR, TIL exhibited cytotoxic activity against K562 cells, a typical target for NK cells. In contrast, TIL from the patient that failed to respond to the treatment showed no activity against these cells. One could speculate that the presence of cells with NK activity could help the TIL anti-tumour responses [39]. 

 Adoptive immunotherapy leads to clinical responses in some HGG patients: Objective responses have been observed with the minimal number of 3 × 10^8^ LAK cells and 10^7^ CTL injected intracerebrally. The absence of clear correlation between the number of effector cells, their cytotoxic activity against autologous tumours, and the clinical outcome might be due to the large variability observed between patients and the limited number of patients enrolled in these trials. This renders difficult, if not impossible, to define an optimal scheme of treatment based on published clinical trials using adoptive immunotherapy. Injection of CTLs or TIL appeared however to allow higher objective responses compared to LAK. Using a mathematical model, Kronik et al. have predicted that GBM would be eradicated by intensive doses from 3 × 10^8^ to 2 × 10^9^ alloreactive CTL injected every 4 to 5 days, dependent of the size of the tumour burden [45]. The impact of chemotherapy or corticosteroids on the treatments' efficacy is also controversial. Whereas these drugs were completely avoided in some trials because of their immunosuppressive effects [23, 31, 33, 34], others studies have shown no influence of steroids or chemotherapy on the generation and the lytic activity of the effector cells [24, 26, 27]. Remarkably, the eosinophil infiltration at the tumour site and in CSF is described in several clinical trials using LAK and CTL [30, 32, 38, 39]. This might be linked to the use of IL2 or GM-CSF, known to induce systemic hypereosinophilia. However, the involvement of these cells in the anti-tumour response should be addressed.

## 3. Active Immunotherapy

 Several antigen sources can be used for active immunotherapy such as intact tumour cells, tumour protein lysates, tumour-derived mRNA, peptides eluded from tumour MHC class I molecules, and synthetic peptides. Antigens can be used alone and injected in the presence of different adjuvants or presented on DCs which play a key role in initiating the immune reaction.

### 3.1. Active Immunotherapy Using ATC

Vaccines based on ATC have been used for HGG in eight studies (Table 3) [46–53]: five pilot studies of antitumour vaccination [47, 48, 50–52], one phase I trial [53] and reported in two cases report [46, 49] for a total of 71 treated patients. ATCs are generally inactivated by radiation, sometimes genetically modified [46, 49, 51], and can be infected with a virus [47, 50] to boost the induced immune reaction. In one case, the cells harvested after surgery were treated with antisense oligonucleotides for insulin growth factor receptor 1 (IGF-IR/AS ODN) before implantation [48]. One pilot study used ATC collected from a fixed tissue, which enabled inclusion of a greater number of patients [52]. Indeed, the establishment of primary lines is a limiting factor; the lack of enough tissue can retard the first vaccination after surgery.

 The cells were administered subcutaneously in six series, and intradermally in two others. Injections were associated in two studies with IL2 [47] or GM-CSF infusions [49]. The number of cells injected varied from 10^6^ to 10^11^. The vaccination was repeated in all of these studies, generally for three cycles with a total of 1 to 13 injections. 

 Tolerance was acceptable in all trials, without any grade III/IV toxic events. There were only a few cases of fever, erythema, and perturbed liver tests. Preventive measures for thromboembolism had to be associated with IGF-IR/AS ODN administration [48].

 In most studies reported, at least some patients exhibited immune response induction either in periphery (DTH against the injected tumour cells or anti-tumour responses of PBMC) or most importantly at the tumour site. CD8 T cell infiltration was found for example in one study in six out of seven patients who underwent a reoperation for relapse, but was not found in the four control patients who had a reoperation [50].

 The clinical response was evaluated radiologically in five studies demonstrating 4 CR (3 GBM), 6 PR (4 GBM), 2 MR (2 GBM), and 6 SD (6 GBM) in a total of 53 patients.

 Three patients with SD in the study of Clavreul et al. appeared to have a longer than usual survival (42, 62, 88 weeks) after a second surgery without other treatment [53]. In this particular study, GM-CSF was infused continuously or discontinvously at the site of the ATC inoculation using a programmable pump. Such a longer survival was also the case for one GBM patient in another study [51]. Steiner et al. [50] found a better PFS in 23 GBM treated patients (40 weeks) in comparison with a control group of 87 patients (26 weeks). These authors demonstrated a benefit in terms of OS (100 versus 49 weeks), 1-year survival (91% versus 45%), 2-year survival (39% versus 11%), and even 3-year survival (4% versus 0%).

### 3.2. Active Immunotherapy Using Dendritic Cells

Vaccination with DC has been the most widely studied option: 19 studies (Table 4) [54–72], 11 phase I trials [55, 56, 59–61, 63, 64, 66, 67, 70, 71], 5 phase I/II trials [57, 62, 65, 69, 72], one phase II trial [68], and two case reports [54, 58] including 313 patients. The antigen sources were varied: tumour lysates, peptides eluded from ATC, defined peptides, mRNA derived from ATC, and whole ATC in 10, 4, 1, 1, and 4 studies, respectively. For vaccinations using ATC, the ATC were fused or incubated with DC. Defined peptides were derived from EGFRvIII [70], which appears to be a particularly interesting target as its expression is frequent in GBM [13] and specific of malignant cells. 

 In most studies, DC were prepared using a standard method, that is, from peripheral monocytes cultivated in the presence of GM-CSF and IL4. In six studies, they were matured using different cocktails with IL1*β*, TNF*α*, PGE2, or IFN*γ* [57, 61, 63, 71, 72]. In one case, a TLR agonist (OK432) was used [65]. Vaccines were injected intradermally or subcutaneously. In one study, some patients also received intracerebral injections [57]. In one, the vaccination was combined with injections of recombinant IL12 [55]. The number of cells injected ranged from 10^6^ and 10^10^. The number of injections and the frequency of the injections were also highly variable.

 Tolerance appeared to be acceptable with only one grade IV neurotoxicity (stupor) event being reported for all of these studies [65]. This patient with stupor had a large residual tumour and a perilesional edema probably induced by vaccination. Most of the toxic effects were grade II (headache, seizure, flu-like syndrome). A peripheral immune response (demonstrated by *in vitro* tests or DTH) was observed in more than half of patients. For the twenty-four patients who relapsed after vaccination and had an analysable tumour, lymphocyte infiltrations, particularly CD8 cells, were found in 15 patients. 

 The radiological response was described in 10 studies with 6 CR (4 GBM), 11 PR (5 GBM), 7 MR (6 GBM), and 27 SD (14 GBM) among the 130 patients. Thirteen studies have reported a beneficial effect in terms of survival compared with historical cohorts or nonrandomized control groups [56, 57, 59, 61, 62, 64, 65, 67–72]. Two studies were unable to find any correlation between induced peripheral immune response and clinical response [64, 72]. From the largest cohort of patients reported (34 GBM), Wheeler et al. showed however that responders presented a global increase of IFN*γ* synthesis (before versus after vaccination using *in vitro* PBMC stimulation) compared with non-responders. In addition, this study showed that responders to vaccination exhibited a better response to chemotherapy delivered in a second phase [68]. In their study, Liau et al. [64] had four patients with increased intratumoural infiltration by lymphocytes who had been vaccinated at a time when the tumour was minimal. This T-cell infiltration was correlated with decreased intratumour TGF*β* and with better survival. The opposite was observed for three other patients without T-cell infiltration (vaccination in presence of a major tumour, OS < 12 months, and strong presence of intratumour TGF*β* at relapse). One study showed that patients vaccinated with mature DC had better survival than those vaccinated with immature DC and that conjoint administration of DC in the peripheral blood and intracranially gave a superior response than peripheral injection alone [65].

 Summarizing, active immunotherapy appears to have a beneficial effect in some patients, particularly those with a small tumour, without major toxicity. Both clinical trials using ATC and DC demonstrate induced immune responses (detected by DTH, tumour infiltration and/or anti-tumour responses of PBMC) and some clinical responses. Once again, due to the large variability of protocols tested in these trials, that is, the source of ATC, the type of DC used, the cell number injected, the number of injection, the type of adjuvants, it is almost impossible to recommend a particular approach. One can just point out that for DC, no dose-related toxicity or efficacy has been demonstrated [64]. In addition, it seems better to favour mature DC compared to immature DC.

## 4. Discussion

 There were 76 objective responses (18 CR and 58 PR) in the 396 HGG patients described in the trials reported in this paper. Forty-nine and 27 responses were reported for adoptive and active immunotherapy, given objective responses rates of 23% and 15% respectively. Only patients with residual disease at the time of the vaccination can be evaluated for CR or PR; patients who undergo a gross total resection can only achieve stable disease as their best radiographic outcome. For other solid tumours, similar criteria based on radiological tumour measurements (RECIST-based criteria) are used. Using these criteria, a 3.3% overall objective response rate was reported for 1306 patients with different metastatic cancer vaccinated with peptides, pox viruses, tumour cells, or dendritic cells [73]. Higher response rate seems therefore to be observed for HGG. However, one must emphasize that some trials reported in this paper do not specify the exact criteria to define a PR and/or the duration of the response. To achieve a CR or PR according to Macdonald criteria, the most widely used criteria for assessing responses to therapy in HGG, the radiological response must be sustained for at least 4 weeks. Furthermore, clinical assessment and corticoid dose must also be taken into account. Few reports are currently evaluated following these criteria, which renders difficult comparisons between trials, but could also lead to an optimistic interpretation of some of them explaining the very high percentage of responses. If clinical response has to be evaluated following an immunotherapy protocol, one should advise to use the Macdonald criteria, in particular the last recommendations issued from the international neuro-oncology working group that take into account some limitations of these criteria [74].

 The interaction between injected or induced immune cells and tumour cells can lead to an equilibrium rather than a destruction. Objective criteria based on changes in the tumour masses may therefore not be well adapted to assess this type of response. That is why survival is often regarded as a better endpoint after immunotherapy. Several studies reported in this paper have shown better survival for patients treated with immunotherapy compared to patients from historical cohorts or nonrandomized groups. All are phase I/II trials, with a limited number of patients, which must lead to a cautious interpretation. Lower tumour grade, extent of resection, younger age, good performance status, mutation of IDH1, as well as an intact neurological function are recognised as favourable prognostic factors for HGG [75]. These factors should therefore be taken into account for the survival analysis. This implies the implementation of trials with higher numbers of patients and/or more selected patients to lead to more conclusive data about the impact of immunotherapy for HGG and GBM patients.

 The immune therapy was in these trials well tolerated, with few grade III/IV adverse events. Cerebral oedemas were often detected in active immunotherapy using effector cells directly infused in the tumour site. However this side effect is difficult to separate from oedema usually observed after surgery. Alternatively, IL2 and GM-CSF used as adjuvant, might lead to vascular perturbations leading in edema formation. Therefore, an MRI before this type of treatment is necessary to distinguish the increased cerebral edema due to surgery from immunotherapy. The lack of other major side effects could be explained by the selectivity of the treatment aiming at destroying specifically tumour cells. Alternative explanations could be the relative inefficacy of the treatment or the frequently use of steroids in glioma that could prevent some major side effects. Up to now, major toxicity has not been reported, except for one patient with bulky residual tumour, for whom an overwhelming peritumoural inflammatory reaction was observed, after injection of DCs [61]. Phase III will help to validate the positive impact on survival, without severe adverse event.

 Treatment for newlydiagnosed GBM begins with surgery when feasible, followed by focal radiotherapy (RT). For patients up to 70 years, the current standard of care comprises also a temozolomide (TMZ) chemotherapy during and after RT. At recurrence, repeat surgery, as well as new lines of chemotherapy like nitrosourea or bevacizumab (+/− irinotecan) may prolong survival in some patients. Despite this multimodal treatment, mean overall survival remains below 16 months [76]. Most studies reported in this paper began while TMZ was not included in the standard care, allowing the choice of immunotherapy immediately after RT in newlydiagnosed patients. In some patients, TMZ has marginal activity, if any, and MGMT status is a strong predictive factor of response to TMZ [77]. These patients should therefore benefit from alternative strategies, like immunotherapy. Before this, the best technique for MGMT analysis has to be assessed [78]. Another solution is to add immunotherapy to the standard treatment, as recently reported for 8 patients for whom DC injections were performed between the end of the radio/chemotherapy and the beginning of the adjuvant chemotherapy [72]. This is based on the hypothesis that combination of treatments could increase the tumour-specific response, as it has been observed in animal models. RT could kill tumour cell by apoptosis, favouring cross-presentation by APC, and it has been shown that it upregulates the expression of MHC molecules by tumour cells favouring their killing by CTL [79–81]. TMZ could decrease the number of regulatory T cells; also this effect was only observed with a low-dose metronomic regimen in a SC-implanted rat glioma model [82]. CCL2 is produced by glioma tumour cells and has both direct and indirect inhibitory effects, among which attraction of T reg cells. As TMZ can reduce CCL2 secretion by glioma tumour cells, this could augment indirectly the efficacy of immunotherapy [83, 84]. Currently, most of the proposed immunotherapy trials for HGG required that patients are operated on, either to perform direct intracranial injection, or to get tumour as source of tumour antigens. This induces a limitation in the number of patients who can be enrolled in this type of trial, particularly at the time of recurrence (less than 10% have a second surgery in the study of Bauchet et al. on 952 GBM patients [76]). Another potential limitation comes from the corticosteroids given to patients to reduce tumour-associated oedema. Dexamethasone, frequently taken by patients before and sometimes after surgery induces a deep decrease in peripheral lymphocytes, as well as an increase of abnormal circulating monocytes characterised by the phenotype CD14+/HLA-DR low/neg [85]. This could prevent the obtaining of large number of effector cells in some adoptive approaches and therefore decrease the treatment efficacy, although the impact of corticosteroids is not clear for the reported studies. Furthermore CD14+/HLA-DR low/neg monocytes are unable to fully differentiate into mature DC, which can be a problem for vaccination with DC. That is why some trials are restricted to patients off steroids at the time of leukapheresis and during vaccination. 

 Up to now, the best results for patients with metastatic melanoma have been obtained with adoptive therapy involving lymphodepletion followed by the intravenous injection of *exvivo* expanded TIL plus IL2. The addition of the lymphodepletion preparative regimen consisting in a chemotherapy (cyclophosphamide and fludarabine) and whole body irradiation resulted in objective response rates of 52% and 72% in two trials with both 25 patients, with increased survival compared to patients who received T cells without lymphodepletion. One of the key improvements is the persistence of infused cells, which is highly associated with objective response. Lymphodepletion could act by eliminating competition for homeostatic cytokines, in particular IL15, for which high levels can be detected only after treatment. Other factors, like elimination of T reg cells could also play major roles [86]. In case of residual tumour after surgery, adoptive cell therapy could therefore be an option for HGG, but it has to be refined in order to increase efficacy. The direct intracerebral delivery by infusion in the tumour site at the time of surgery or by the use of catheter/reservoir systems is often chosen in immunotherapy using LAK and CTL cells. Indeed, this particular route bypasses the finely regulated migration through the BBB and therefore allows to have high number of effector cells at the tumour site. Data comparing with the same cells peripheral and local injection are lacking, but clinical results are also observed after intravenous injection. Standard *in vitro* tests, such as phenotyping of effector cells or killing assays against tumour cells, should help to predict the treatment efficiency on a particular patient. However, the phenotype of the effector cells used in the different clinical trials with adoptive therapies varies between patients and has no clear impact on the clinical outcome that is, the presence of T-LAK (CD3+) and LAK cells (CD3− CD56+) in LAK trials or the ratio of CD4/8 cells in CTL trials. Moreover, most of the studies using LAK and CTL cells could not correlate the high cytotoxicity activity of the effector cells observed *in vitro* against autologous tumour cells and the *in vivo* efficiency of the treatment. This highlights the need to integrate the recent advances in the field in order to improve the quality of the injected cells. As the killing activity of LAK is not specific, it seems better to use CTL. CTL can be expanded from TIL repeatedly stimulated *in vitro*. This technique works very well for melanoma, a tumour wellknown to induce significant numbers of CTL during the natural course of the disease. Though Quattrocchi et al., in the only trial using this approach, expanded up to 3 × 10^9^ lymphocytes from tumours [35] it could be less adapted for glioma patients. Another common source of CTL is PBMC from the patient. Up to now, published studies have used autologous tumour cells as antigenic stimulations, which can lead to polyclonal expansion of specific CD8 and CD4 cells. Over the last decades, significant progress in active immunotherapy has been achieved in melanoma patients owing to the discovery of well-defined tumour antigens expressed on melanoma cells. As several glioma-associated antigens (GAAs) are now known, it becomes possible to expand CD8+ T cells against these GAA, giving the possibility to treat patient even when tumour cells are not available, but also allowing genetic modifications of the autologous lymphocytes [87]. As persistence of cells *in vivo* is a major point for the success of adoptive immunotherapy, it is interesting to note that persistence of effector cells at the site of the tumour was often transient as observed on biopsies or tumour samples analysed after relapse in the reported studies. In the addition to improve the injected cells, working on this point could therefore lead to great improvements. Instead of proposing heavy treatments inducing lymphodepletion, the use of alternative cytokines to IL2, like IL15 could be tested as well as the combination of an active vaccination. In an animal model, the concomitant adoptive transfer of T cells and a vaccination improves substantially the anti-tumour efficacy of the treatment [88]. It is likely that all these developments will soon benefit to adoptive T-cell therapy against malignant gliomas. 

 Most studies suggest that cancer vaccine could have the most benefit in state of low tumour burden. This is also observed in a series of 56 recurrent GBM patients vaccinated with DC. Total resection before vaccination was the only predictor of a better survival [69]. In contrast to adoptive immunotherapy, for which administered effector cells can lead to a direct lysis of tumour cells, active immunotherapy requires the initiation and development of an immune reaction. Due to the risk of a rapid regrowth of the tumour before the development of an effective antitumoral reaction in case of residual disease, one may advice treatment with active immunotherapy only after maximal tumour resection. 

 Vaccination strategy can consist of inoculation of inactivated autologous tumour cells. The first limitation step of the approach is the culture of the tumour cells. It is not always possible to establish primary cell lines from malignant gliomas. In the study of Parney, on 116 malignant gliomas, culture was successful in 61% of cases [51]. In some cases *in vitro *growth is not sufficient to allow vaccination. The delay between the beginning of the culture and the time when vaccine preparation is available can be very long. Median expansion times range between 4.5 and 30 weeks [47, 50, 51, 53], which explain that some patients may progress before receiving the vaccine. One additional limitation is the possibility that the immunologic phenotype of glioma cells might change after passaging *in vitro* (Anderson, 2002) [89], which could decreases the efficacy of the vaccine. Very often, cells are injected with adjuvants like IL2 or GM-CSF are modified to express costimulatory molecules or cytokines, or are infected with virus in order to increase their immunogenicity. The way all these potential adjuvants act is poorly understood and their real impact is debate. For example, the use of combined GM-CSF is supported by numerous preclinical studies, in gliomas and in other cancers [90–92]. However, two recent randomized trials in melanoma patients showed that GM-CSF could be harmful as immune adjuvant. In the first study, patients received allogeneic tumour cells and BCG, with or without GM-CSF. A diminished DTH response as well as an increase in early deaths was observed in the GM-CSF arms [93]. In the second study, 12 MHC class I-restricted and one HLA-DR-restricted tetanus peptides were injected with or without GM-CSF. CD8+ and CD4+ T-cell response rates to the injected peptides were higher without GM-CSF [94]. Effects could be different according to the route of administration and the doses [45]. A possible mechanism by which GM-CSF could interfere is the induction and activation of myeloid-derived suppressor cells [95]. This highlights well that manipulating the immune system can induce stimulation as well as suppression. All the difficulty of immunotherapy lies in finding the good equilibrium between both.

 Most of vaccination protocols for HGG patients have used DC, the most potent APC. This reflects the popularity of this vaccine approach: up to now, more than 3000 patients suffering from varied cancers, enrolled in more than 200 trials have received DC (http://www.mmri.mater.org.au/). Many recent reviews have focused on the use of DC as cancer vaccine [96–98], and in particular for malignant gliomas [99–101]. Many options are available about the way to prepare DC, to load them with tumour antigens, to inject them. Some processes used in the protocols for HGG patients require to have autologous tumour cells, to elute peptides, or to fuse them with DC. This implies large cultures of tumour cells and thus the problems already discussed. To overcome this issue, glioma-cell lysate are used in a number of trials. This has the advantage of providing a panel of personalised class I and class II peptides, like with autologous tumour cells. There is however a theoretical risk of autoimmune encephalomyelitis, due to the frequent presence of nontumour glial cells in the tumour. Though up to now such side effects have not been reported, one can fear that in the case of higher efficacy of immunotherapy it may happen. DC can be loaded with synthetic tumour antigens like peptides (short or long), proteins or transfected with encoding antigen nucleic acids. In one published study, DC were pulsed with EGFRvIII peptides [70] and in an ongoing study, DC are loaded with 4 peptides derived from EphA2, IL-13R*α*2, YKL-40, and GP100 [18]. The use of defined TAA reduces the risk of autoimmunity (if TAAs are specific to tumour cells), but it implies the previous selection of patient according to their MHC haplotype if using peptides and sometimes patients are also selected according to the expression of the tumour antigens by the tumour. Currently, a universally expressed glioma TAA does not exist, limiting the usefulness of this approach to patients whose tumour expresses the selected TAA. Furthermore, with the use of a limited number of tumour antigens, there is a theoretically greater risk of an immune escape due to clonal expansion of antigen-loss variants. Among the improvement expected in the loading of DC, the use of antigens expressed by tumour stem cells could be a promising perspective, due to the major role these cells may play in the regrowth of tumour after treatment [102, 103].

 About the best way to generate DC *in vitro*, specialists consider that mature DCs (mDC) are superior to immature DCs (imDC) [98]. They can migrate in higher rate to the draining lymph nodes after peripheral injection, they upregulate several costimulatory molecules and can produce different cytokines. Improving the maturation protocol, in order to have the “perfect” DC remains a big challenge in the field. Most published clinical trials in HGG have used imDC or DC matured with pro-inflammatory cytokines. An interesting alternative to mature DC is the use of Toll-like receptor (TLR) ligands, which can mimic bacterial or viral infections, triggering a process of maturation in DC. In the study of Yamanaka et al., patients received either imDCs or DCs matured with OK432, a streptococcal preparation acting notably through the activation of TLR4. Patients vaccinated with mDC had longer survival than those vaccinated with imDC [65]. Van Gool et al. reported an improvement in PFS curves between HGG whose skins were pretreated with imiquimod (which binds TLR7) before the injection of DC matured with TNF*α* and IL1*β* and patients receiving only DC matured with TNF*α*, IL1*β*, and PGE2 [99]. The maturation cocktail designed by Mailliard and colleague (poly I:C, IFN*α*, TNF*α*, IL1*β* and IFN*γ*) allows the generation of fully mature DC, with high migratory responsiveness to lymphoid organ chemokines and an ability to produce significant levels of IL12p70 after CD40 ligation (which mimics the interaction between DC and CD40L-activated cells in lymph nodes) [104]. In a model of tumour-bearing mice such so-called *α*-type-1 polarized DCs, pulsed with peptide antigens can migrate into draining lymph nodes after SC injection and induce antigen-specific CTL, allowing a prolong survival. The secretion of CXCL10 by these DC seems to play a major role in the induction of specific CTL and their homing to the brain [105]. As the efficacy of type-1 *α*-DC loaded with glioma-associated antigen epitopes, in combination with the administration of poly-ICLC, is currently tested in recurrent malignant gliomas [18], the translation (or not) of these promising results into the clinic will be known soon. 

 The relevance of intravenous DC injection has never been tested for HGG patients. It is worth noting that the only autologous cellular immunotherapy approved by the US FDA is based on the intravenous administration of PBMC cultured with recombinant PAP-GM-CSF for the treatment of metastatic prostate cancer [106]. In most of the studies reported in this paper, DC were injected in the skin. As priming of T cells within the cervical lymph is important to induce effector T cells with the ability to home in to the central nervous system, injection of DC in regions allowing them to reach these nodes appears to be a good option. Direct intranodal injection, under echographic guidance is also feasible and allows more DC to reach the nodes. However, following intranodal injection, DC are not always correctly injected into the nodes [107, 108] and as limited numbers of DC in the draining lymph nodes are sufficient to induce a specific immunologic response [109], intradermal injections appear as the best compromise. In their study, Yamanaka et al. showed that patients receiving both intratumoral and intradermal DC injections had longer overall survival than those receiving only intradermal DC [65]. The usefulness of this combination has recently been confirmed in animal models: the addition of intratumour injection to SC injections leading to an increase of CD8+ T cells in the tumour and an increase in survival [105, 110]. Intratumoural DC activity could be due to intratumour secretion of cytokines and chemokines (depending on the type of DC injected) and/or migration of DC to draining lymph nodes. A better understanding of this mechanism should therefore lead to considering this option for future trials. 

Whatever the immunotherapy approach, one fundamental element to take into consideration is the corrupted tumour microenvironment which favors tumour development. Different mechanisms developed by glioma cells can suppress anti-tumour immune responses. Several defects at the systemic level have been reported in glioma patients such as decreased T-cell responsiveness [111, 112], increased circulating regulatory T cells [113, 114], and defective monocytes and DC functions [115–117]. Various factors produced by glioma cells might contribute to these defects such as TGF*β*, PGE2, and IL10 [118–123]. 

 Beside these systemic effects, glioma cells develop several molecular strategies to inhibit directly the immune cell effectors at the tumour site. Several intracellular, membrane and soluble molecules have been described as taking part in this phenomenon such as CD70 [124, 125], FasL [126–128], gangliosides [125], HLA-G [129], PDL-1 [130–132], IDO [132–134], and TGF*β*1-3 [135]. We have recently described the involvement of TGF*β*2, IDO, and PDL-1 on GBM immunosuppressive properties on T-cell functions in a tumour-specific T-cell model [132]. Different groups have also focused on immunosuppressive cells that are recruited at the site of the tumour. Indeed, tumour immune-derivation involved a particular chemokine profile that could specifically induce the recruitment immunosuppressive cells such as T reg and myeloid-derived suppressor cells (MSDC). Glioma-infiltrating microglia/macrophages (GIM) represent the largest population infiltrating human glioma with around a third of the cells present in the tumour mass [114]. GIM are poor T-cell activators due to lower expression levels of molecules such as HLA class II or costimulatory CD80 and CD86 molecules [114]. Furthermore, GIM secrete factors that support the tumour invasion and proliferation [18]. As described systemically, T cells are also present at the tumour site [114, 136, 137], although their presence seems to have no impact in patient prognosis. MDSC are a heterogeneous population of cells that can suppress T-cell responses [138]. One recent study performed in a rat model has shown an infiltration of such cells following immunization with tumour antigens [139]. These cells could therefore play a major role in gliomas.

 A better efficacy could certainly associate optimized standard immunotherapy protocols with treatments designed to hamper this immunomodulation. The early results using immunomodulator molecules have been encouraging (as an example CTLA4 blockade in melanoma patients [140]) and one trial in melanomas recently demonstrated the potential interest of combination of CTLA4 blockade with a DC vaccine [141]. Different strategies are currently under study to decrease the deleterious effect of Treg as injection of low-dose cyclophosphamide (metronomic cyclophosphamide) [142], or injection of CD25 (high) targeting immunotoxin (denileukin diftitox). This latter approach has also been tested in association with DC vaccination [143]. Molecules designed to inhibit the action of TGF*β*, IDO, and PD1 are also being tested clinically. Preclinical studies in glioma models have shown the interest to combine vaccination with inhibition of TGF*β* [144] or depletion of Treg [145].

 In conclusion, immunotherapy can be considered as a potential new interesting weapon for treatment of patients with HGG and one should expect many improvements of this type of approach over the next years. However, there is a need for well-designed trials with higher numbers and more homogenous patients, taking into account the current recognized prognostic or predictive factors like and leading to phase III randomized studies.

## Figures and Tables

**Table 1:** Adoptive immunotherapy in high-grade gliomas using lymphokine activated killer (LAK) cells*.

References	Type of trial	Patients	Administration	Clinical responses
[23]	Phase I	*N* = 6 progressive HGG	IC (1 injection)	No PR or SD

[24]	Phase I	*N* = 13 recurrent GBM	IC (2 injections)	1 SD No survival benefit

[25]	Phase I	*N* = 9 recurrent HGG 7 GBM and 2 AA	IC (15 injections)	1 PR (AA) No survival benefit

[26]	Phase I	*N* = 20 recurrent HGG 11 evaluated: 9 GBM/2 AA	IC (1 to 2 injections)	Median survival after IT: 18 weeks (>90 weeks—*N* = 2) Median OS: 63 weeks *Correlation between survival and the number of LAK injected *

[27]	Phase I/II	*N* = 19 recurrent gliomas14 GBM/5 GIII	IC (2nd injection if survival >4 months)	2 PR (GIII) 2 PR, one after 2nd injection (GBM) Median survival after IT: 30 weeks*No correlation between clinical and radiographic responses *

[28]	Pilot study	*N* = 5 recurrent GBM	IC (1 injection)	No PR or SD No survival benefit

[29]	Phase I	*N* = 9 recurrent GBM	IC	1 CR, 2 PR, 4 SD Median OS: 18 months

[30]	Phase I/II	*N* = 19 recurrent HGG 15 GBM, 4 AA	IC (2 injections) 2nd cycle if no PD	1CR (AA), 1 delayed CR, 2 PR, 1SD (GBM) Median survival after IT: 53 versus 25.5 weeks (GBM)

[31]	Phase I	*N* = 10 recurrent malignant tumours 4 GBM, 2 AIII-IV, 2 AII-III, 1 AOA, 1 MDB	IC (*N* = 9)Intrathecal (*N* = 1 MDB)(4 to 5 injections)	2 PR (AII-III) Median survival after IT: 13 weeks Median OS: 78 weeks

[32]	Phase I/II	*N* = 28 recurrent HGG	IC (2 injections) 2nd cycle if no PD	1 CR (AA) 1 CR, 2 PR (GBM) Median OS: 53 versus 26 weeks (GBM)

[33]	Phase I/II	*N* = 40 recurrent GBM	IC (1 injection)	Median survival after IT: 9 months (excluded secondary GBM) Median OS 17.5 versus 13.6 months (excluded secondary GBM)

[34]	Phase I/II	*N* = 33 newly diagnosed GBM post RT+CT	IC (1 injection)	Median OS: 20.5 months *Correlation between survival and the number of LAK injected; Correlation between survival and absence of corticoids before treatment *

*: Abbreviations used in this table: AII/III: grade II and III astrocytoma; AIII/IV: grade III and IV astrocytoma; AA: anaplastic astrocytoma; AO: anaplastic oligodendroglioma; AOA: anaplastic oligoastrocytoma; CR: complete response; CT: chemotherapy; GII/III: grade II and III glioma; GIII/IV: grade III and IV glioma; GBM: glioblastoma; HGG: high-grade glioma; IC: intracranial injection; IT: immunotherapy; MDB: medulloblastoma; OS: overall survival; PR: partial response; RT: radiotherapy; SD: stable disease.

**Table 2:** Adoptive immunotherapy in high-grade gliomas using cytotoxic T lymphocytes (CTL) or tumour-infiltrated lymphocytes (TIL)*.

References	Type of trial	Patients	Administration	Immune response	Clinical responses
CTL obtained from PBMC					

[35]	Phase I	*N* = 5 HGG 2 newly diagnosed GBM, 2 recurrent GBM, 1 AOA	IC (7 to 13 injections)		2 PR (1 GBM, 1 AOA) No survival benefit (survival >2 years— *N* = 1 AOA)

[36]	Phase I	*N* = 4 HGG 3 GBM, 1 AA	IC (3 injections)		3 PR (1 AA), 1 SD

[37]	Phase I	*N* = 10 recurrent HGG 7 GBM, 2 AA, 1 AOA	IC (3 injections)		1 CR (AA), 4 PR (3 GBM, 1 AA), 3 SD (GBM) Median survival: > 5 months

[38]	Phase I	*N* = 5 recurrent HGG 2 GBM, 1 AA, 2 AO	IC (3 to 11 injections)	Cerebrospinal fluid hypereosinophilia	3 SD (1 AA, 2 AO) No survival benefit

CTL obtained by lymphocytes from draining lymph nodes or PBMC after vaccination with irradiated ATC					

[40]	Phase I	*N* = 15 recurrent HGG 12 GBM, 3 AA	IV (1 to 3 injections)	DTH (15/15)	No PR or SD Free disease survival: ≥ 8 months (*N* = 7) (>40 months *N* = 1)

[41]	Phase I	*N* = 10 recurrent HGG 9 GBM, 1 AA	IV (1 injection)		3 PR (1 AA, 2 GBM) Survival after reoperation: > 1 year (*N* = 4/8)

[42]	Pilot study	*N* = 9 recurrent HGG 6 GBM, 3 GIII	IV +/− intracarotid infusion (1 injection)	DTH (9/9)	3 PR (1 GBM, 2 GIII with survival > 4 years) *Correlation between clinical response and CD4/CD8 composition of infused cells. *

[43]	Phase I	*N* = 12 newly diagnosed glioma: 6 GBM, 2 GII, 4 GIII	IV (1 to 2 injections)		4 PR (2 GBM, 2 GIII) 2 SD (2 GII)

[44]	Pilot study	*N* = 19 recurrent HGG16 GBM, 2 AA, 1 gliosarcoma	IV (1 injection)	DTH (17/19)	1 CR, 7 PR, 9 SD Median survival: 12 months *Correlation between survival and DTH response *

Autologous TIL					

[39]	Pilot study	*N* = 6 HGG 3 GBM, 3 AA	IC (2 injections)		1 CR (AA), 2 PR (1 AA, 1 GBM)

*: Abbreviations used in this table: see Table 1; DTH: delayed-type hypersensitivity; PBMC: peripheral blood mononuclear cells; IV: intravenous.

**Table 3:** Active immunotherapy in high-grade gliomas using autologous tumour cells (ATC)*.

References	Type of trial	Patients	Antigen source Immune activation	Administration	Immune response	Clinical responses
[46]	Case report	*N* = 1 recurrent GBM	irradiated ATC + fibroblasts genetically modified to secrete IL 2	SC (10 injections)	ATR PBMC (lytic activity)	No survival benefit at 4 months

[47]	Pilot study	*N* = 11 newly diagnosed GBM after surgery and RT	ATC infected with NDV and inactivated with cisplatinum	SC (4 to 5 injections)	DTH (11/11-infected ATC)DTH (3/11-ATC) TI CD4/CD8 T cells (4/4)	No survival benefit*No correlation between survival and DTH response *

[48]	Pilot study	*N* = 12 progressive HGG8 GBM, 4 AA	ATC + IGF-IR/AS ODN	SC (1 to 10 injections)	TI lymphocytes (4/9)	1 CR 2 PR 2 SD (GBM) 1 CR, 2 PR (AA)

[49]	Case report	*N* = 1 recurrent GBM	irradiated ATC + fibroblasts genetically modified to secrete IL 4	ID (2 injections in 5 sites)	No ATR PBL (ELISPOT)	1 PR-survival: 10 months

[50]	Pilot study	*N* = 23 GBM after RT	irradiated ATC infected with NDV	ID (5 to 8 injections)	DTH (15/15) ATR PBMC (3/3) (IFN*γ* ELISPOT) TI CD8 cells (6/7)	1 CR Median OS: 100 versus 49 weeks

[51]	Pilot study	*N* = 63 recurrent GBM 3 melanoma	irradiated ATC transduced with B7-2 and GM-CSF	SC (3 injections)	No ATR PBMC(CTL activity) (GBM)	Longer free disease survival (3/6–1 GBM)

[52]	Pilot study	*N* = 12 GBM 8 newly diagnosed GBM 4 recurrent GBM	formalin-fixed ATC tuberculin microparticles as adjuvant	SC (3 injections in 5 sites)	DTH (9/12)	1 CR, 1 PR, 2 MR, 1 SD Median survival: 10.7 months 3 of 5 responders survival > 20 months

[53]	Phase I	*N* = 5 recurrent HGG 4 GBM, 1 AOA	irradiated ATC	SC (4 injections )	DTH (2/5)	3 SD (GBM)

*: Abbreviations used in this table: see Table 1 and Table 2; ATR: anti-tumour responses; ID: intradermal injection; IGF-IR/AS ODN: insulin-like growth factor type I receptor antisense oligodeoxynucleotide; MR: minor response; NDV: Newcastle-Disease-Virus; PBL: peripheral blood lymphocytes; SC: subcutaneous; TI: tumour infiltration.

**Table 4:** Active immunotherapy in high grade gliomas using dendritic cells (DCs)*.

References	Type of trial	Patients	Antigen source	DC maturation	Administration	Immune response	Clinical responses
[54]	Case report	*N* = 1 recurrent GBM	Acid eluted peptides from allogenic GBM	none	ID (3 injections)	TI T cells	No survival benefit

[55]	Phase I	*N* = 8 progressive HGG 5 GBM, 2 AA, 1 AO after RT and/or CT	Fusion of ATC with DC	none	ID (1 to 7 injections)	Increased CD56 in PBL (4/5) ATR PBMC (6/6) (IFN*γ* ELISA)	1MR, 4SD (GBM) 2SD (AA, AO)

[56]	Phase I	*N* = 9 newly diagnosed HGG: 7 GBM, 2 AA after RT	Antigens eluted from ATC	none	ID (3 injections)	ATR PBMC (4/7) (lytic activity) TI CD4, CD8, CD45RO cells (2/4)	Median survival after IT: 455 versus 257 days (GBM)

[57]	Phase I/II	*N* = 10 HGG 7 GBM after RT,3 recurrent GIII	ATC lysates+ KLH	none	ID and/or IC (1 to 10 injections)	DTH (3/6) Increased CD56 (5/5); CD8, CD16, CD19 (4/5) in PBLATR PBMC (2/5) (IFN*γ* ELISPOT) TI CD4, CD8 cells (2/2)	2 MR, 2SD (GBM), 2SD (GIII) OS > 200 weeks (*N* = 2 ID + IC; *N* = 1 ID)

[58]	Case report	*N* = 1 recurrent GIII	ATC lysates	none	ID (6 injections)	DTH after 2nd vaccination	CR maintained 2 years after IT

[59]	Phase I	*N* = 14 HGG 1 GBM, 1 AA, 9 recurrent GBM, 3 recurrent AA	ATC lysates	none	SC (3 injections)	Increased IFN*γ*RNA in PBMC (6/10) ATR T cells (4/9) (HER-2, gp100, MAGE-1 tetramers) TI CD8, CD45RO cells (3/6)	Median survival after IT: 133 versus 30 weeks (8 recurrent GBM)

[60]	Phase I	*N* = 7 relapsed brain tumours 2 GBM, 1 AA, 4 others Age: < 25 years	ATC RNA	none	ID (3 to 5 injections)	No ATR PBMC (0/3) (IFN*γ* ELISA)	1 PR (1XA) 4 SD (1AA, 3 others)
[61]	Phase I	*N* = 12 recurrent HGG 11 GBM, 1 PXA	ATC lysates	IL1*β*, TNF*α*, and PGE2	ID (2 to 7 injections)	DTH after 2 vaccinations (6/8) DTH after 5 vaccinations (7/8)	1 PR, 1 SD,1 CR (GBM) 1 CR (PXA) Median OS: 10.5 months 36-month OS: 17%

[62]	Phase I/II	*N* = 25 newly diagnosed GBM after RT Vaccine alone (*N* = 12) Vaccine and chemotherapy (*N* = 13)	ATC lysates or peptide elutions	none	ID (3 injections)	Vaccine alone: ATR PBMC (4/11) Vaccine and chemotherapy: ATR PBMC (4/13) (lytic activity and IFN*γ* Q-PCR)	Vaccine or chemotherapy alone: 2-year survival: 8% Vaccine and chemotherapy: 3 PR - 2-year survival: 42%

[63]	Phase I	*N* = 15 recurrent HGG 6 GBM, 7 AA, 2 OAA	Fusion of ATC with DC	TNF*α*	ID (3 injections)	DTH (15/15) ATR PBL (2/8) (lytic activity)	1 SD (GBM), 3 PR, 1 MR (AA)1 PR, 1SD (AOA)

[64]	Phase I	*N* = 12 GBM 7 newly diagnosed GBM 5 recurrent GBM	Acid elution from ATC	none	ID (3 injections)	ATR peripheral T cells (6/12) (lytic activity) TI CD8 CD45RO cells (4/8)	1 PR Median OS: 23.4 versus 18.3 months

[65]	Phase I/II	*N* = 24 recurrent HGG 18 GBM, 6 GIII after RT+nitrosourea	ATC+ KLH	none or OK432	ID (immature or matured DC) +/− IC (immature DC) (1 to 10 injections)	DTH (8/17) ATR PBMC (7/16)(IFN*γ* ELISPOT)	1PR, 3MR, 6SD (GBM) 4SD (GIII) Median OS: 480 versus 400 days *Longer survival if DC maturation or IC injection *

[66]	Phase I	*N* = 2 HGG 1 recurrent GBM, 1 recurrent AAafter total resection	Irradiated ATC	Fibroblast transduced TGF/IL4	ID (2 injections)	ATR PBMC (1/1) (EphA2 ELISPOT)	2 PR
		*N* = 5 HGG 5 newly diagnosed GBM after total resection+RT	ATC lysates	Mixture of TGF/IL4 transduced fibroblasts with DC (IFN*γ*, IL1*β* and TNF*α*)	ID (2 injections)	No response	No response

[67]	Phase I	*N* = 139 GBM (2 recurrent), 4 AA (3 recurrent)	inactivated ATC	none	ID (2 to 13 injections)	TI CD8 CD45RO T cells (3/3)	12-month survival: 46% 18-month survival or more: 23%

[68]	Phase II	*N* = 34 GBM 23 recurrent 11 newly diagnosed	ATC lysates	none	ID (3 to 4 injections)	ATR PBMC (17/34) (IFN*γ* PCR)	3 CR, 1 PR Median survival after IT: 642 days versus 430 days

[69]	Phase I/II	*N* = 56 recurrent GBM	ATC lysates	IL1*β*, TNF*α* and PGE2	ID (3 to 9 injections +/− ATC lysates)	DTH (9/21 at diagnostic, 2/12 after vaccination)	Median OS: 9.6 months

[70]	Phase I	*N* = 12 newly diagnosed GBM	EGFRvIII antigen + KLH	None	ID (3 injections)	DTH EGFRvIII (5/9) DTH KLH (9/9) ATR PBMC (10/12) (EGFRvIII-induced proliferation)	Median OS: 22.8 months

[71]	Phase I	*N* = 45 children HGG 23 GBM, 5 AA, 1 OAO, 16 other HGG	ATC lysates + imiquimod	IL1*β* and TNF*α*	ID (2 to 7 injections) +/− ATC lysates boosts		All HGG: Median OS: 13.5 months GBM alone: Median OS: 12.2 months

[72]	Phase I/II	*N* = 8 newly diagnosed GBM (Stupp protocol)	ATC lysates	IL1*β*, TNF*α*, and PGE2	ID (4 injections+ ATC lysates)	DTH (2/5) Increased CD8/CD25 in PBL (6/7) ATR PBMC (5/8) (IFN*γ* ELISPOT)	Median OS: 24 months

*: Abbreviations used in this table: see Table 1 to Table 3; KLH: keyhole limpet haemocyanin; PXA: pleomorphic xanthoastrocytoma; XA: xanthoastrocytoma.
